# Effects of Housing Systems on Production Performance, Egg Quality, Tonic Immobility and Feather Score in Laying Hens

**DOI:** 10.1002/vms3.70112

**Published:** 2024-11-04

**Authors:** Mert Erek, Erdal Matur

**Affiliations:** ^1^ Department of Physiology Faculty of Veterinary Medicine Van Yuzuncu Yil University Van Turkey; ^2^ Department of Physiology Faculty of Veterinary Medicine Istanbul University‐Cerrahpasa Istanbul Turkey

**Keywords:** housing systems, laying hens, plumage condition scores, production performance, tonic immobility

## Abstract

**Background:**

This study was designed to investigate the effects of different housing systems on production performance, egg quality and welfare in laying hens.

**Methods:**

One hundred and twenty 42‐week‐old “Atak S” laying hens, purchased from a manufacturing company, were randomly assigned to 4 housing systems: conventional cages, furnished cages, deep‐litter system and free‐range. Each system housed 30 hens, which were kept in these systems for 6 weeks. Parameters regarding production performance, egg quality, plumage condition scores and tonic immobility were assessed at the end of the housing period.

**Results:**

Egg production and egg mass were lower in cage‐free rearing systems than in caged systems. Mean egg weight in free‐range hens, and albumen height and Haugh unit in deep‐litter hens, were lower than in other housing systems. Eggshell weight in hens housed in furnished cages was greater than in free‐range hens, while eggshell strength was better compared to that of hens in conventional cages. The housing system did not impact fearfulness; however, the deep‐litter housing increased the sensitivity to touch or capture. Whole body and regional plumage condition scores of free‐range hens elicited more favourable results than those kept in conventional cages. Because the plumage condition indicates welfare, the results proved the superiority of free‐range over conventional rearing regarding welfare.

**Conclusions:**

Concerning the parameters, such as egg production, animal welfare and fear level, overall data revealed the pros and cons of all housing systems investigated. We consider that this study's findings might contribute to the researchers and breeders seeking alternative housing for laying hens.

## Graphical abstract

In cage‐free systems, egg production and egg mass were lower compared to caged systems. Mean egg weight in free‐range hens, and albumen height and Haugh unit in deep‐litter hens, were lower than in other housing systems. Eggshell weight in hens housed in furnished cages was higher than that in free‐range hens, and eggshell strength was superior to that of hens in conventional cages. Housing system did not affect fearfulness, but deep‐litter housing increased sensitivity to capture. Free‐range hens showed superior plumage condition scores, indicating better welfare compared to conventional cages.

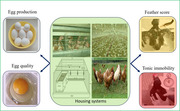

## 1 Introduction

When purchasing an animal product, consumers in developed countries consider not only the product's quality but also the source animal's housing conditions (Mench and Rodenburg 2018) because it is believed that products derived from animals whose metabolic and behavioural requirements have been fulfilled, enabling high comfort, are healthier and more appropriate from the perspective of animal welfare (Vits et al. 2005). This incline in consumers’ demands oriented the breeders to develop alternative production models. Poultry farming is one of the most impacted industrial fields by these innovations. The battery‐type caging system where a massive number of chickens are piled up draws the consumers’ reaction; therefore, facilities with furnished cages, deep litter, free‐ranging areas, or cage‐free rearing systems have become widespread in layer poultry farming (Blatchford 2018). Thus, the pros and cons of the relevant systems have become the topic of many research studies, evaluating several parameters, including product quality, production performance, mortality rate and potential environmental hazards of the housing systems (Şekeroğlu and Sarıca 2005).

Some researchers reported that egg production performance was higher in caged hens than in cage‐free systems (Tumova and Ebeid 2003; Voslarova et al. 2006). On the contrary, vice versa was also accurate for free‐range compared to caged systems (Yıldırım and Kaya 2017). As for the manufacturer's aspect, high production performance is not adequate, and that the product is delivered to consumers with the least possible loss is also of great importance. Hence, massive post‐production losses were previously noted due to insufficient eggshell resistance (Ketta and Tůmová 2017); therefore, the potential effects of applications in laying‐hen husbandry on eggshell quality have drawn the attention of the researchers. Eggshell strength was previously shown to be higher in conventional cages than in furnished cages and the deep‐litter system (Ledvinka et al. 2012; Englmaierová et al. 2014). However, a recent study suggests that eggs from free‐range hens may exhibit greater resistance to breakage, indicating that the relationship between housing systems and eggshell strength is more complex than previously understood (Alig et al. 2023).

Consumers tend to believe that eggs produced in cage‐free systems are of better quality (Vits et al. 2005). Some researchers supported this postulate. Likewise, Rodríguez‐Hernández, Rondón‐Barragán, and Oviedo‐Rondón (2024) reported that the Haugh unit of the eggs of cage‐free systems was higher than that of caged systems. Contrasting data is also available, documenting a higher Haugh unit in the eggs of conventional cages than in other housing systems (Englmaierová et al. 2014).

The immobility test is utilized to estimate welfare and fear status in chicken farming (Rentsch et al. 2023). The duration of tonic immobility was reported to be shorter in cage‐free hens, provided with more enhanced facilities than in caged systems (Campbell et al. 2022). Likewise, the duration of tonic immobility was shorter in furnished cages than in conventional cages (Hrabcakova et al. 2012). Nevertheless, on the other hand, no significant difference was noted among different housing systems concerning tonic immobility (Yilmaz Dikmen et al. 2016).

Feather quality is an indicator of welfare in poultry (Hüttner et al. 2023), which offers a great deal of understanding of the housing conditions, including the birds’ interactions and health status (Pichová et al. 2017). Researchers presented diverse data regarding the plumage condition in the hens of different housing systems. Blatchford et al. (2016) stated that free‐range hens exhibited more favourable plumage conditions than caged birds. Sherwin et al. (2010) showed that feather quality was higher in the deep‐litter hens than in conventional cages. On the other hand, according to Yilmaz Dikmen et al. (2016), neck feather loss in conventional cages was slightly more than in furnished cages, yet the total feather score remained unchanged.

To sum up, ambiguity and incoherency have been noted in the previously documented data concerning egg production performance, egg quality and animal welfare in different housing systems (Philippe et al. 2020), and despite the conducted studies, whether or not the eggs of cage‐free systems are of higher quality than conventional and furnished cages sustains its vagueness (Racevičiūtė‐Stupelienė et al. 2023). Therefore, further studies are required to elucidate the topic.

This study's aim was to evaluate the potential effects of different housing systems on production performance, egg quality, tonic immobility and plumage condition.

## 2 Materials and Methods

### 2.1 Hens and Experimental Design

Forty‐two‐week‐old 120 “Atak‐S” laying hens were involved in the study. All hens were randomly distributed into 4 groups: conventional cages, furnished cages, deep‐litter system and free‐range, and each group contained 30 birds. The hens in the conventional and furnished cages were allocated into six subgroups, each containing five birds. No subgroups were constituted in the deep litter and free‐range due to the nature of these systems. All hens were allowed for 2 weeks to adapt to the new environmental conditions and recover from transfer‐associated stress; therefore, the experimental procedures were initiated at the 44th week and ceased at the 49th week of age. Standard vaccination program and beak trimming had been performed before the hens’ transfer to the facility.

### 2.2 Housing Systems and Conditions

Hens in the conventional cage system were housed in six battery‐type metallic wire cages with the dimensions of 50 × 60 × 56 cm^3^ (width × length × height), each containing five birds; thus, an area of 600 cm^2^ was occupied per hen. The cages contained feeders and nipple drinkers.

The furnished cage system involved six wire cages with the dimensions of 63 × 240 × 50 cm^3^ (width × length × height), and five hens were housed in each cage, allowing a minimum space of 3024 cm^2^ per hen. The cages were furnished with feeders, nipple drinkers, a laying area of 30 × 40 × 40 cm^3^ (width × length × height) sectioned by a dark‐coloured plastic curtain, a rugged plastic ground‐scratching platform of 40 × 40 × 1 cm^3^ (width × length × height), pecking strings composed of white coloured fibred nylon and two perches composed of cylindrical metal bars placed parallelly and 10 cm above the ground. The system's environmental conditions, such as temperature, humidity and lighting, were adjusted according to the instructions proposed for “Atak‐S” rearing. Minimum, maximum and mean temperature and relative humidity values in conventional and furnished cages were estimated at 13°C, 18°C and 15.2 ± 1.62°C and 53%, 81% and 67.7% ± 9.28%, respectively. Because the cages were equipped with an automatic air conditioning system, they were not affected by the climate changes in the outer environment. The hens were kept under a 14:10 h light/dark cycle during the experimental procedures.

A chick‐rearing poultry house with a base area of 160 m^2^ and ventilation, lighting and heating facilities was used to create a deep‐litter system, providing a space of 5.34 m^2^ per hen. The house's floor was covered with 15‐cm‐thick wood shavings, and the roof was rigged with adequate hanging feeders and drinkers. One‐meter‐long wooden cylindrical bars were erected 25 cm above the floor and 20 cm away from the walls to construct perches providing a perching space of at least 20 cm per hen. Moreover, one wooden nesting box was placed per every five hens. In the deep‐litter system, the minimum, mean and maximum temperature were 13°C, 19°C and 16.1 ± 1.52°C, whereas the minimum, maximum and mean relative humidity were measured as 54%, 85% and 69.3% ± 7.8%, respectively.

The free‐range rearing system involved an indoor poultry house and a fenced yard with a roaming space of 500 m^2^. The indoor facility with the base of a flat floor contained perches and stationary nesting boxes like the deep‐litter system. The gate opening to the yard was left open during the daytime, allowing hens to roam freely. Moreover, adequate feeders and drinkers were placed in the indoor house and the yard, enabling the hens’ ad libitum access to water. The minimum, maximum and mean temperature and relative humidity values were recorded as 3°C, 27°C and 13.3 ± 4.81°C and 55%, 99% and 85.0% ± 10.6%, respectively, according to the meteorological data on the climate estimated during the experimental period.

### 2.3 Feeding of the Hens

All hens were fed commercial chicken feed. The amount of feed consumed per day was assessed according to the “Atak S” rearing manual. The feed's energy, protein levels and nutrient composition are shown in Table 1. Feeding was provided by an automatic system in the conventional and furnished cages, whereas the hens in the deep‐litter and free‐range systems were fed manually once a day in the morning between 08:00 and 10:00 AM.

**TABLE 1:** Nutrient composition of the basal diet.

Feed ingredients	%
Soybean meal (44% CP)	34.0
Sunflower seed meal	26.0
Full‐fat soybeans	11.0
Maize	15.0
Soybean oil	0.98
Dicalcium phosphate	2.30
DL‐Methionine	0.12
Limestone	9.60
Vitamin + mineral premix^a^	1.00
Salt	0.30

Calculated analysis	
Crude protein	17.29
Crude fat	3.85
Crude fibre	2.7
Ash	15.15
Methionine	0.31
Methionine + cystine	0.52
Lysine	0.69
Threonine	0.46
Calcium	3.69
Available phosphorus	0.43
ME, MJ/kg feed	10.96

*Note*: ME, MJ/kg food = (0.03431 × crude fat, g/kg) + (0.01551 × crude protein, g/kg) + (0.01669 × starch, g/kg) + (0.01301 × sugar, g/kg).

^a^ Vitamin + mineral premix = vitamin + mineral content per 1 kg feed: vitamin A 10,000,000 IU; vitamin D3 2000,000 IU; vitamin K3 3 mg/kg; vitamin B1 3 mg; vitamin B2 6 mg: vitamin B6 4 mg; vitamin B1 2 mg; 15 mg; Ca pantothenate 10 mg; niacin 25 mg; folic acid 1 mg; biotin D 25 mg; Mn. 80 mg; Fe 60 mg; Zn 60 mg; Cu 5 mg: Co 500 mg; Se 150 mg.

### 2.4 Production Performance

Eggs in each housing system were collected and weighed every morning between 08:00 and 10:00 AM. A 0.1 g high‐precision digital weighing scale (XT 6200C, Precisa, Switzerland) was used for the measurements. Egg production, mean egg weight and egg mass were calculated according to the following formulas. Egg production rate = the number of produced eggs/the number of hens ×100, and the percentage value of daily egg production per hen was determined. Mean egg weight was calculated by the equation: mean egg weight = egg weight/the number of eggs. Egg mass = (egg production rate × mean egg weight)/100 (Keten and Matur 2022).

### 2.5 Egg Quality Parameters

Effects of different housing systems on egg quality parameters were assessed on the last eggs collected in the 49th week. After being held for 24 h, 30 eggs were randomly selected from each group and analysed (conventional cage; *n* = 6 × 5, 30 eggs in total, furnished cage; 6 × 5, 30 eggs in total, deep‐litter housing; 30 eggs in total and free‐range; 30 eggs in total). A digital egg quality tester (Digital egg tester, DET6000, Nabel, Co. Ltd., Japan) was used to analyse the internal and external egg quality parameters. All analyses were conducted in a private enterprise's research and development laboratory (SEN Agriculture & Industrial Inc., Bandırma, Balıkesir).

External quality parameters, such as egg weight (g), egg/eggshell ratio (%), eggshell thickness (mm) and eggshell strength (kgf), were measured. Eggshell weight (g) was assessed by a precision scale. The eggshell ratio was calculated by the following equation: eggshell ratio = (eggshell weight × 100)/egg weight).

Haugh unit, albumen height (mm) and yolk colour were assessed as internal quality parameters. The Haugh unit was calculated by the following formula: HU = 100 × log (H‐1.7W^0.37^ + 7.6), (HU = Haugh unit, H = thick albumen height, W = egg weight).

### 2.6 Tonic Immobility Test

Tonic immobility was tested on the last week (49th week) of the study. Eighteen hens selected from each group were subjected to the testing. The selection of 3 hens from each subgroup of conventional and furnished cages was randomized (3 × 6 = 18 hens). Hens tested were marked with plastic collars to avoid evaluation reiteration.

The testing process was standardized for each group by being performed by the same experimenter under constant conditions in a separate enclosed room. Utmost care was paid to avoid stress induction in hens. The testing was initiated by positioning the hen in the right lateral recumbency. While gently holding the bird on the head and neck with one hand, light pressure was applied laterally with the other, enabling the bird's immobility. The hen was restrained in this position for 15 s and slowly released. Then, the experimenter moved 30 cm away from the bird, ensuring not being seen. The instant the hen has moved his head or any body part, the “time to the first movement,” and the instant it has stood up, the “time to righting” after immobilization, were recorded by a chronometer (Fogelholm et al. 2019).

### 2.7 Scoring Plumage Condition

The plumage condition was scored according to the method proposed by Tauson (1984) on the study's last week (49th week). Twenty randomly selected hens from each group were involved in the scoring. Six body parts, such as the head, neck, back, wing, tail and chest, were included for regional feather status evaluations. Initially, the body regions were photographed, and the pictures were transferred to a computer screen. The same researcher who also performed the shooting scored the plumage and integumentary conditions on these images. The plumage condition was scored on a 1–4 scale as follows: 4 = favourable plumage; almost no feather and integumentary damage; 3 = moderate plumage condition; although some feathers were regionally damaged, the whole integument was covered with feathers; 2 = fair plumage; marked regional feather damage, with respectively small (<5 cm) patches of featherless skin on the evaluated body regions, and 1 = poor plumage; feathers were severely damaged, with large patches (>5 cm) of featherless skin. The total score was calculated by the sum of each regional score. Because 6 body regions were scored, the minimum and maximum scores were 6 (6 × 1 = 6) and 24 (6 × 4 = 24) per hen, respectively.

### 2.8 Statistical Analyses

An SPSS program (Version 11.5.2.1, SPSS Inc., Chicago, IL, USA) was used for statistical analyses of the data. The Shapiro–Wilk test was initially applied to determine whether or not the data were normally distributed. Those below a *p* value of 0.05 were recognized as non‐normally distributed data. Skewness and Kurtosis values were also taken into account while interpreting the distribution of the data. The data of the normally distributed parameters were compared by the variance analysis (ANOVA). When the ANOVA exerted a significant difference between the groups, the Tukey HSD test was applied as the post hoc test. For non‐normally distributed data, whether or not there was a difference between the groups was analysed by the non‐parametric Kruskal–Wallis test. When significant, the pairwise comparisons were performed by the Mann–Whitney *U*‐test to determine the differences between groups. Because the data concerning total and regional feather scores and tonic immobility were not normally distributed, the non‐parametric Kruskal–Wallis test was applied for the group‐wise statistical analyses of these parameters. The Mann–Whitney *U*‐test was performed for pairwise comparisons when the difference was significant. Statistical significance was established at a *p* value of 0.05, and the values between *p* = 0.05 and *p* = 0.1 were recognized as a trend. The overall data were presented as mean values ± standard error.

## 3 Results

**FIGURE 1:**
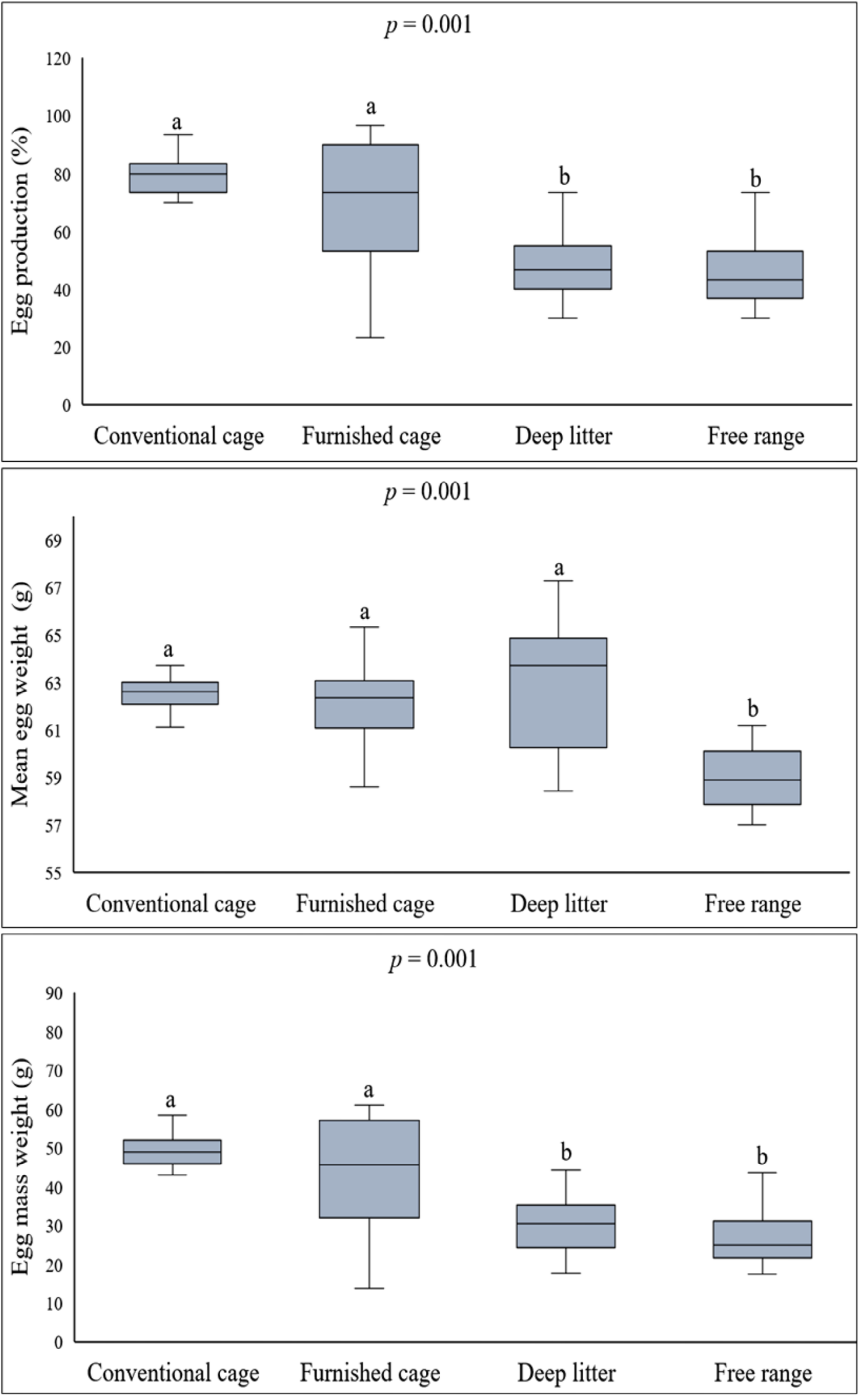
The effect of housing systems on production performance. The central lines in the box plots represent the median, the edges of the boxes represent the interquartile range, the whiskers represent the maximum and minimum values. *n* = 120 (there are 30 hens in each group). a and b = The difference between groups with different letters is significant (*p* < 0.05).

Egg production performance and egg mass in conventional and furnished cages are higher than in deep‐litter and free‐range houses (*p* = 0.001 and *p* = 0.001, respectively). Free‐range hens’ mean egg weight was lower than those reared in other housing systems (*p *= 0.001) (Figure 1).

Egg weight in free‐range housing was decreased compared to the conventional cage system (*p* = 0.017). On the other hand, no significant differences regarding the relevant parameter were noted in furnished cages and deep‐litter housing compared to the conventional cage system. Eggshell weight in furnished cages was increased compared to free‐range (*p* = 0.008). No significant differences were recorded among groups regarding eggshell ratio and eggshell thickness (*p* = 0.100 and *p *= 0.143, respectively). The data concerning eggshell strength revealed the superiority of furnished cages over the conventional cage system in terms of resistance to breakage, yet no significant differences were noted when compared with the other housing systems (*p* = 0.017) (Table 2).

**TABLE 2:** The effects of different housing systems on external egg quality parameters in 42‐week‐old laying hens.

	Egg weight (g)	Egg shell weight (g)	Eggshell ratio (%)	Eggshell thickness (mm)	Eggshell strength (kgf)
	Mean ± SE	Mean ± SE	Mean ± SE	Mean ± SE	Mean ± SE
Conventional cage	64.3^a^ ± 0.7	7.7^ab^ ± 0.13	12.0 ± 0.19	0.32 ± 0.05	3.1^b^ ± 0.1
Furnished cage	63.0^ab^ ± 0.7	7.9^a^ ± 0.14	12.6 ± 0.22	0.33 ± 0.05	3.7^a^ ± 0.2
Deep litter	63.4^ab^ ± 0.9	7.7^ab^ ± 0.11	12.2 ± 0.17	0.34 ± 0.05	3.3^ab^ ± 0.1
Free‐range	60.2^b^ ± 1.3	7.2^b^ ± 0.18	12.0 ± 0.18	0.33 ± 0.07	3.2^ab^ ± 0.2
*p* values	0.017	0.008	0.100	0.143	0.017

*Note*: The difference between means with different letters (a and b) in the same column is significant (*n* = 30).

Abbreviation: SE, standard error.

Haugh unit and albumen height of the hens housed in the deep‐litter system were lower than those in the other housing systems (*p* = 0.001 and *p* = 0.001). The egg yolk was darker in colour in the free‐range than the other groups (*p* = 0.001) (Table 3).

**TABLE 3:** The effects of different housing systems on internal egg quality parameters in 42‐week‐old laying hens.

	Haugh unit	Albumen height (mm)	Yolk colour
	Mean ± SE	Mean ± SE	Mean ± SE
Conventional cage	84.6^a^ ± 1.24	7.4^a^ ± 0.20	5.1^b^ ± 0.12
Furnished cage	81.1^a^ ± 0.91	6.8^a^ ± 0.14	5.5^b^ ± 0.13
Deep litter	73.1^b^ ± 1.77	5.7^b^ ± 0.22	4.2^b^ ± 0.32
Free‐range	83.4^a^ ± 1.62	7.1^a^ ± 0.26	8.3^a^ ± 0.23
*p* values	0.001	0.001	0.001

*Note*: The difference between means with different letters (a and b) in the same column is significant (*n *= 30).

Abbreviation: SE, standard error.

**FIGURE 2:**
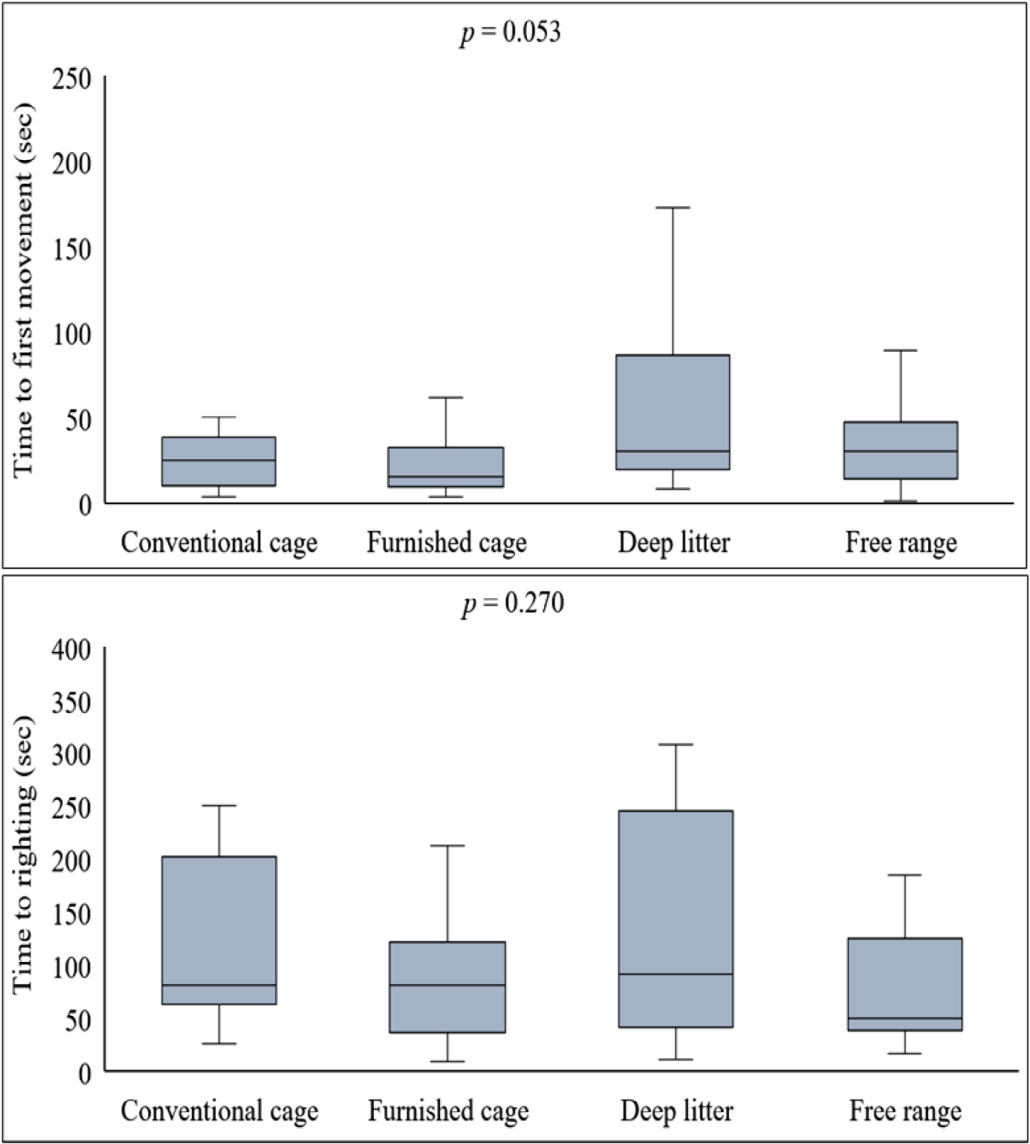
The effect of housing systems on tonic immobility test. The central lines in the box plots represent the median, the edges of the boxes represent the interquartile range, the whiskers represent the maximum and minimum values, and the dots represent outliers. *n* = 72 (there are 18 hens in each group).

The data regarding tonic immobility are presented in Figure 2. No statistically significant difference was noted among groups regarding the relevant parameter; nevertheless, time to the first movement tended to extend in the deep‐litter system (*p* = 0.053). No significant differences were noted among groups regarding time to righting (*p* = 0.270).

The data regarding plumage condition scores are presented in Table 4. The head feathers in furnished cages were in more favourable condition than in conventional cages (*p* = 0.024). The neck feathers of the free‐range hens were in the most favourable condition, followed in descending order by those of the deep‐litter house, furnished cages and conventional cages, respectively (*p* = 0.001). Regarding back feathers, free‐range had the highest scores, followed in descending order by furnished cages, the deep‐litter house and conventional cages, respectively (*p* = 0.001). Wing feathers were in the most favourable condition in free‐range hens. The furnished cages and deep‐litter house scores were equivalent, and conventional cages had the lowest scores (*p* = 0.003). Considering tail feathers, free‐range hens were in better condition than in conventional and furnished cages. On the other hand, no statistically significant difference was noted between the free‐range and deep‐litter housing systems regarding the relevant parameter (*p* = 0.001). The chest feathers of the deep‐litter hens were in more favourable condition than those of conventional cages. The differences between deep‐litter housing and the other two systems regarding the chest plumage condition were statistically insignificant (*p* = 0.005). The overall plumage scoring revealed that the plumage of free‐range hens was prominently in more favourable condition than those of conventional cages. Nevertheless, the differences between free‐range and the other two housing systems were statistically insignificant (*p* = 0.001).

**TABLE 4:** The effect of different housing systems on feather scores in 42‐week‐old laying hens.

	Conventional cage	Furnished cage	Deep litter	Free‐range	
	Mean ± SE	Mean ± SE	Mean ± SE	Mean ± SE	*p* value
Head	2.7^b^ ± 0.21	3.5^a^ ± 0.20	2.9^ab^ ± 0.24	3.3^ab^ ± 0.16	0.024
Neck	2.4^c^ ± 0.20	2.7^bc^ ± 0.23	3.3^ab^ ± 0.23	3.7^a^ ± 0.11	0.001
Back	1.7^c^ ± 0.24	2.8^ab^ ± 0.22	2.1^bc^ ± 0.30	3.3^a^ ± 0.16	0.001
Wing	2.7^b^ ± 0.14	2.8^b^ ± 0.21	2.8^b^ ± 0.21	3.6^a^ ± 0.14	0.003
Tail	2.6^b^ ± 0.23	2.8^b^ ± 0.23	3.1^ab^ ± 0.21	3.7^a^ ± 0.13	0.001
Chest	2.6^b^ ± 0.20	3.2^ab^ ± 0.19	3.6^a^ ± 0.14	3.3^ab^ ± 0.18	0.005
Total	14.9^b^ ± 0.77	18^ab^ ± 0.82	17.8^ab^ ± 0.98	21.1^a^ ± 0.35	0.001

*Note*: The difference between means with different letters (a–c) in the same row is significant (*n* = 18). The feather condition of the chickens was scored from 1 to 4 points: (1) the affected body region is entirely featherless or has very few feathers; (2) a significant deterioration of the feathers and large areas of featherless skin on the affected body region; (3) the plumage was damaged, but the affected body region is still covered with feathers; (4) whole‐body feathers are in a favourable condition.

Abbreviation: SE, standard error.

## 4 Discussion

In the study, the reduced egg production in uncaged systems (Figure 1) was associated with increased energy consumption due to a more active life and conversion of dietary energy intake mainly for physical activities instead of egg production, which had been previously reported (Meng et al. 2015). Additionally, in uncaged housing systems, the likelihood of consuming materials with low energy levels found in the litter material or free‐ranging area was previously suggested to have played a role in reduced egg production (Philippe et al. 2020). Furthermore, it is known that the hens’ cracking or even eating the eggs in uncaged housing systems reduces production performance (Englmaierová et al. 2014).

In previous studies, the mean egg weight in uncaged systems was lower than in caged housing (Dong et al. 2017; Sharma et al. 2022). Likewise, in our study, the mean egg weight was lower in free‐range compared to the two caged systems. On the other hand, interestingly, the egg weight of the free‐range hens was also lower than the other uncaged system (deep‐litter housing). Such a decrease was associated with the ambient temperature, which can be kept under control in the deep‐litter housing (temperature = min 13°C, max 19°C and mean 16.1 ± 1.52°C) but not in free‐range. Hence, low (Li et al. 2020) and high (Getabalew and Negash 2020) ambient temperatures were reported to have adversely impacted egg weight. Increasing energy consumption under low and reduced feed consumption and high ambient temperatures causes a decrease in egg weight (Hughes, Dun, and McCorquodale 1985). When the overall data were evaluated, the mean egg weight of the free‐range was lower than the other three groups (Figure 1). However, it should be noted that this difference had gradually subsided in the samples collected on the final week to assess egg quality parameters, and a statistical significance was determined only in comparison with the conventional cages (Table 2). Hens develop adaptive responses to environmental conditions, mainly temperature, with ageing (Abioja and Abiona 2021). Considering that egg weight is adversely affected by environmental factors, the adaptive responses of the ageing hens might have contributed to the increase in the egg weight of the final week's egg samples, which explains the equivalence in the egg weight values except for those of conventional cages.

In laying‐hen farming, the egg mass is a more specific parameter than the egg production rate and the egg weight in assessing production performance because it involves both the number of eggs produced and the mean egg weight (Alaşahan, Akpinar, and Bozkurt 2013). In this study, the egg mass values of caged systems were higher than uncaged housing systems (Figure 1), which was also reported in previous studies comparing the caged systems with the deep‐litter housing (Abo Abo Ghanima et al. 2020; Voslarova et al. 2006). This phenomenon was earlier mentioned to be associated with high energy consumption levels in uncaged systems. However, diverse data indicated no difference between the caged and uncaged systems regarding the relevant parameter (Peric et al. 2007). Moreover, in contrast, some authors reported that the egg mass values of the free‐range (Yilmaz Dikmen et al. 2016) and deep‐litter houses were higher than those of the caged systems (Soomro et al. 2019). These conflicting data might have resulted from several factors, including differences in diet, environmental conditions, or breed‐wise diverseness; yet, the potentially influential factors should be further investigated to elucidate the precise parameters.

The eggshell quality has substantial significance in the commercial egg production industry. The eggshell is supposed to endure several processes, including the laying phase, collection via automated belts, packaging and transportation. The eggshell quality is crucial for protecting the egg content against potential contamination, and poor shell quality increases the risk of bacterial contamination (Vlčková et al. 2018). Several parameters are considered in assessing the eggshell quality. In this study, we measured the eggshell weight, thickness, rate and strength to determine the potential effects of different housing systems on eggshell quality.

The data revealed that the eggshell weights of the free‐range hens were lower than those of furnished cages. Similarly, Vlčková et al. (2018) reported a decrease in the eggshell weights of free‐range hens compared to those housed in furnished cages. On the other hand, previous studies indicated the ineffectiveness of the housing system on the eggshell weight (Kralik et al. 2013; Kühn et al. 2014). The eggshell rate should be considered to better understand the effects of different applications on the eggshell weight because the eggshell weight is directly correlated with the egg weight. Therefore, the shell rate was calculated on the basis of the egg and eggshell weights. Our data regarding the eggshell rate revealed no statistically significant differences among the housing groups (Table 2), which was compatible with the previous studies (Van Den Brand, Parmentier, and Kemp 2004; Cerolini, Zaniboni, and La Cognata 2005).

In the study, the housing system did not affect the eggshell thickness (Table 2), which is among the essential parameters indicating the likelihood of the unharmed delivery of the egg to the consumer; however, it should be concurrently evaluated with the eggshell strength. After all, the eggshell thickness is one of the components to calculate the shell resistance (Vogel 2003). We determined that the eggs of the furnished cages were more cracking resistant than those of the conventional cages (Table 2). Likewise, Jones, Karcher, and Abdo (2014) reported that cracking resistance was higher in the eggs of the hens housed in the furnished cages. On the other hand, there are conflicting data concerning eggshell resistance. Although Van Den Brand, Parmentier, and Kemp (2004) reported that the eggshells of the uncaged systems were more resistant to crushing, Lichovníková and Zeman (2008) proposed the opposite, indicating that the eggshell resistance was higher in caged systems. On the other hand, no statistically significant differences were noted between the conventional cages and deep‐litter housing (Kühn et al. 2014) or the free‐range (Yenice et al. 2016). Differences in housing systems were indicated to have impacted the eggshell microstructure (Kulshreshtha et al. 2021) and, thus, the shell strength (Ahmed et al. 2005). However, the underlying mechanism is yet to be elucidated. On the basis of the currently available data, it is not likely to explain why the furnished cage system is superior to the conventional cages in terms of eggshell strength; yet, the main distinction between these two systems is that the furnished cages offer facilities for hens’ natural behavioural expressions reducing the stress level. Hence, our data regarding the heterophil/lymphocyte ratio revealed lower levels in the furnished cages than in the conventional cage system (unpublished data). Stress reduces the absorption of dietary nutrients (Dai et al. 2022), particularly short‐chain fatty acids (van de Wouw et al. 2018), which are known to reinforce eggshell strength (Świątkiewicz et al. 2015). Even though the hen population in our caged systems received the same diet, it was considered that insufficient absorption of dietary short‐chain fatty acids due to high‐stress levels was associated with decreased shell resistance in conventional cages. When the parameters regarding eggshell quality were concurrently evaluated, the furnished cage system revealed a superiority over the other housing systems.

The albumen height provides insight into the egg's freshness and is directly correlated with freshness; the higher the height; the fresher the egg. In this study, the albumen height of the deep‐litter system is lower than those of the other three systems (Table 3). The previous studies also indicated that the relevant value was lower in the deep‐litter hens than in those housed in conventional cages (Roberts 2004; Singh, Cheng, and Silversides 2009). Similar to our findings, no significant difference was noted between the caged systems and free‐range regarding the albumen height (Kralik et al. 2013; Kucukkoyuncu et al. 2017).

Haugh unit is the most significant internal egg quality parameter indicating the egg's protein quality and freshness (Williams 1992). In the study, the Haugh unit of the deep‐litter eggs was markedly lower than those of the other three housing systems (Table 3). The main difference between the deep‐litter and free‐range or caged systems is the ammonia accumulation issue. In the deep‐litter system, the litter material stores ammonia, continuously exposing the hens to this chemical (Singh, Cheng, and Silversides 2009). Ammonia at high concentrations impairs the egg white composition by impacting the pH (Minelli et al. 2007), eventually reducing the Haugh unit. Accordingly, low Haugh unit in the deep‐litter eggs was associated with ammonia accumulated in the litter. Hence, we may deduce that the deep‐litter system is disadvantageous concerning protein quality and freshness of the eggs compared to the other systems investigated.

Dietary carotenoid content determines egg yolk colour (Roberts 2004): The higher the carotenoid, the darker the yolk colour (Rossi and De Reu 2011). There is no direct correlation between the egg's nutrient value and the egg yolk's yellow colour (Dvořák et al. 2007). Nevertheless, high carotenoid content in eggs elected for laying contributes to the anti‐inflammatory system, enabling the healthy development of the offspring (Surai and Speake 1998). In the study, free‐range eggs had a markedly darker yellow colour (Table 3). Previous studies also reported that free‐range eggs had darker yellow yolk (Hammershøj and Steefedt 2005; Sokołowicz, Krawczyk, and Dykiel 2018). Diverse findings are available regarding yolk colour; however, this diversity was indicated to have resulted from dietary supplements or the plant vegetation the hens had access to instead of the housing system (Kucukkoyuncu et al. 2017). In‐line with these data, Karadas et al. (2005) also determined that free‐range eggs had high carotenoid content. Therefore, we may deduce that free‐range eggs are more expedient to fulfilling consumers’ yolk colour demand.

The tonic immobility test determines fearfulness levels in poultry (Ferrante et al. 2009). In this study, although no differences were noted among the housing systems concerning the time to righting, the time to the first movement tended to rise in the deep‐litter system (Figure 2). Compatible with our findings, the duration of tonic immobility was shown to have extended more in the deep‐litter hens than in the caged systems (Anderson and Adams 1994). It was also reported that, apart from the housing system, several factors, including transportation, husbandry, management and regular contact with the birds, might have impacted the duration of tonic immobility (Altan et al. 2005).

Herein, we found that the time to the first movement after tonic immobility was longer in the deep‐litter system than in the others (*p* = 0.053). The extended duration was previously reported to be associated with factors such as husbandry, management and the frequency of contact with the birds (Altan et al. 2005). In this study, we considered that the time to the first movement was likely to be affected by the duration to capture the hens because it was previously indicated that when the time to capture the bird was extended, the duration of tonic immobility was also prolonged (Gudev et al. 2011). Capturing uncaged hens is more troublesome and, thus, time‐taking than caged birds. However, of our study's two uncaged systems, the time to the first movement after tonic immobilization was delayed only in the deep‐litter housing, raising the question of why the initial mobility time was not delayed in this system despite the prolonged capturing period. On the basis of our current data, it is a tricky question to answer; nevertheless, this issue was associated with reduced fearfulness in these birds. The main difference between these two uncaged systems is that free‐range hens had free access to consume the vegetation or insects in the roaming area in addition to their diet (Khusro, Andrew, and Nicholas 2012). Insects consumed offer nutrients such as a substantial amount of tryptophan (Bukkens 1997), which was previously reported to have significantly reduced fearfulness in hens (Newberry and Blair 1993). Moreover, a diet supplemented with tryptophan for 15 days shortened the duration of tonic immobility (Gudev et al. 2011). In conclusion, we may deduce that different housing systems did not impact fearfulness levels in birds, yet, increased the sensitivity to capture in the deep‐litter hens.

Plumage condition in laying hens is recognized as a significant indicator of health and welfare (Welfare Quality 2009). In our study, the total feather scoring revealed a more favourable plumage condition in the free‐range system than in conventional cages (*p* = 0.001; Table 4). Feathers are relatively worn off due to the housing conditions in conventional cages (Vasdal et al. 2022). Furthermore, hens in this system have restricted space to express natural behaviours, which induces pecking behaviour (Blokhuis 1986). Birds’ close contact in a confined space adversely affects the plumage condition (Onbaşılar and Aksoy 2005). The free‐range system offers more comfort regarding species‐specific behavioural expressions, such as foraging, dust bathing and preening (Sokołowicz et al. 2020), contributing to a favourable plumage condition (Coton et al. 2019). Moreover, free‐range hens have more access to daylight, which reduces aggressive behaviours such as pecking each other and cannibalism (Spindler et al. 2020). Total feather scores provide insight into plumage condition, and regional feather condition scores should also be considered to assess the underlying causes of feather loss (Campe et al. 2018). In this study, head feathers in furnished cages were in better condition than in conventional cages (*p* = 0.024, Table 4). Conventional caged system hampers the species‐specific behavioural expression in hens, which, in return, emerges as pecking behaviour, causing the birds to hurt each other (Rodenburg 2003). This phenomenon explains the increased head feather loss in conventional cages since pecking behaviour mainly targets the head (Bilcík and Keeling 1999). In a conventional cage system, the birds’ outstretching their necks through the spaces of the wired cages to reach the feeders was shown to have torn the neck feathers (Bishop and Dhaliwal 1994). Feather losses on the back, wings and tail are mainly associated with pecking behaviour (Vasdal et al. 2022). Hence, pecking frequency was reported to be higher in conventional cages (Rodenburg 2003). In our study, chest feathers of the deep‐litter housing were in more favourable condition than in conventional cages (*p* = 0.005, Table 4). Sawdust used as the litter material in the deep‐litter housing system provides more comfort than the caged systems’ wiring. Moreover, the birds’ opportunity to perch during the night was considered to have contributed to a more favourable plumage condition. Hence, the plumage was relatively in better condition in the furnished cages with several perches and in the free‐range compared to the conventional cage system. The wire‐based floor of conventional cages inevitably adversely impacts chest feathers condition. Furthermore, prolonged contact with the cage's front aspect, where the feeders are placed, induces more chest feather loss in conventional cages (Özentürk, Yıldız, and Genç 2022).

## 5 Conclusion

The data regarding production performance parameters revealed the superiority of caged systems over uncaged systems. No difference was noted between the two caged systems in terms of production performance. Nevertheless, considering the positive impact of furnished cages on eggshell strength and the adverse influence of conventional cages on the plumage condition, furnished cages offer more advantages from the breeders’ perspective. We determined that the deep‐litter system adversely affected internal egg quality parameters. Moreover, the hens in this system were more sensitive to touch or capture. Considering the feather scores, it might be concluded that the welfare level of the free‐range was higher, even though the production performance was lower than the other systems.

## Author Contributions

Mert Erek carried out the planning of the study, the conduct of the animal experiments and laboratory analysis. He also contributed to the writing of the article. Erdal Matur contributed to the development of the project idea, organizing the laboratory analysis, interpreting the data and preparing the manuscript. Both of the authors provided critical feedback and contributed to the final manuscript.

## Ethical Statement

All experimental procedures in the study were conducted in accordance with the principles of the Istanbul University Animal Experiments Local Ethics Committee (Approval no: 2018/64).

## Conflicts of Interest

The authors declare no conflicts of interest.

### Peer Review

The peer review history for this article is available at https://publons.com/publon/10.1002/vms3.70112.

## Data Availability

The data that support the findings of this study are available from the corresponding author upon reasonable request.
